# Crystal structure of (−)-(5*R*,7*R*,8*S*,9*R*,10*S*)-8-methyl-7-[(5*R*)-3-methyl-2-oxooxolan-3-en-5-yl]-1-aza-6-oxatri­cyclo­[8.3.0.0^5,9^]tridecan-13-one monohydrate

**DOI:** 10.1107/S2056989018004425

**Published:** 2018-03-27

**Authors:** Takeshi Oishi, Makoto Yoritate, Takaaki Sato, Noritaka Chida

**Affiliations:** a School of Medicine, Keio University, Hiyoshi 4-1-1, Kohoku-ku, Yokohama 223-8521, Japan; b Department of Applied Chemistry, Faculty of Science and Technology, Keio University, Hiyoshi 3-14-1, Kohoku-ku, Yokohama 223-8522, Japan

**Keywords:** crystal structure, isosaxorumamide, tetra­cyclic compound, fused tricyclic core, oxolane, azepane, pyrrolidine, hydrogen bond

## Abstract

The title compound, C_17_H_23_NO_4_·H_2_O, is an epimer of the natural tetra­cyclic alkaloid isosaxorumamide which consists of a fused 5–7–5 tricyclic core and a di­hydro­furan­one substituent. The terminal di­hydro­furan ring is essentially planar with a maximum deviation of 0.0273 (14) Å from the mean plane and oxolane, azepane and pyrrolidine rings in the tricyclic ring system adopt twist, twist-chair and envelope forms, respectively. In the crystal, the amide and water mol­ecules are linked by O—H⋯O hydrogen bonds, forming a tape structure running along the *b-*axis direction. The tapes are further connected by C—H⋯O inter­actions into a three-dimensional architecture.

## Abstract 2

The title compound is an epimer of natural tetra­cyclic isosaxorumamide. The di­hydro­furan­one, oxolane, azepane and pyrrolidine rings adopt planar, twist, twist-chair and envelope forms, respectively. In the crystal, O—H⋯O hydrogen bonds connect the water and main mol­ecules into a tape structure, which is further expanded into a three-dimensional network by C—H⋯O inter­actions.

## Chemical context   

**Figure 1:**
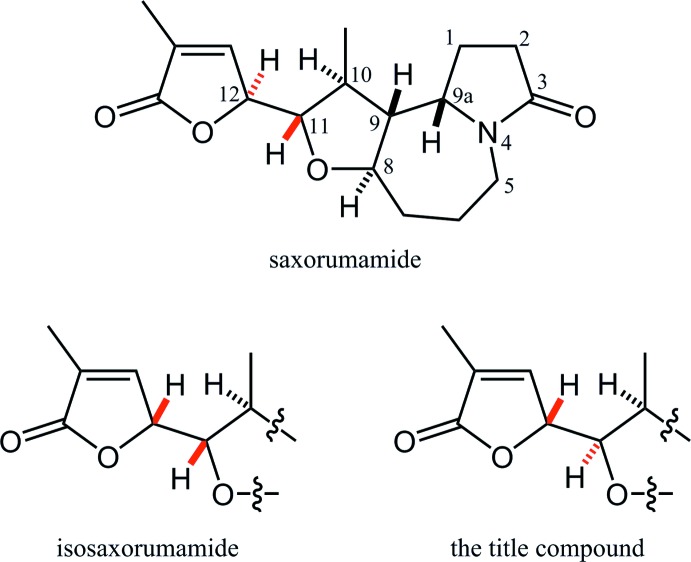
Structures of two natural products, saxorumamide and isosaxorumamide, and the title compound. Differences in the relative stereochemistries of these three diastereomers are shown in red.

Saxorumamide and isosaxorumamide are natural *Stemona* alkaloids isolated from the root of *Stemona Saxorum* (Wang *et al.*, 2007 ▸). They are a pair of diastereomer (12-epimer of each other) which consist of a fused octa­hydro­furo[3,2-*c*]pyrrolo[1,2-*a*]azepane nucleus with a di­hydro­furan­one substituent (Fig. 1 ▸). The *Stemona* alkaloids have been isolated from various *Stemonaceae* species, and over 150 metabolites have been elucidated (Pilli *et al.*, 2000 ▸, 2010 ▸). Extracts of *Stemonaceae* plants have been traditionally used for folk medicines as anti­tussive and anthelmintic agents in the wide regions of East Asia and Southeast Asia (Greger, 2006 ▸). *Stemona Saxorum* has also been utilized for endemic disease in Vietnam. The title compound is an 11-epimer of isosaxorumamide afforded in a synthetic study of stemo­amide-type alkaloids (Yoritate *et al.*, 2017 ▸).
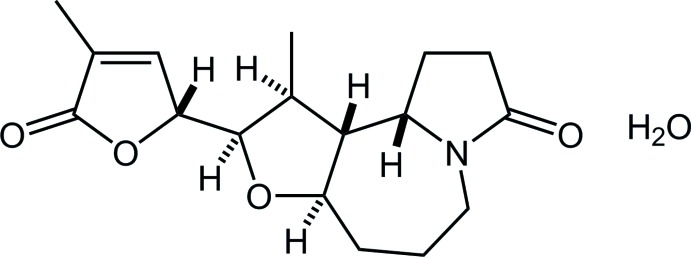


## Structural commentary   

**Figure 2:**
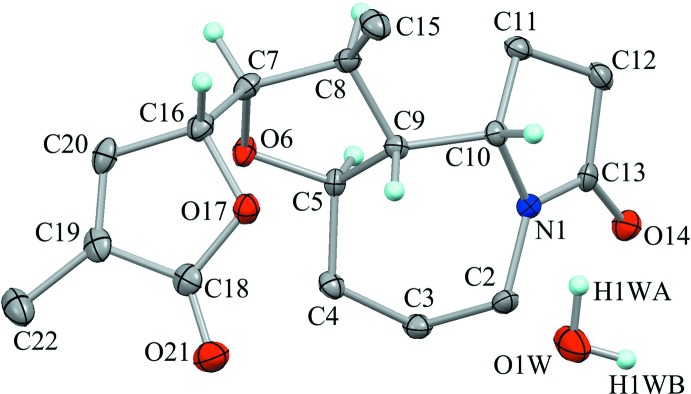
The asymmetric unit of the title compound, showing the atom labelling. Displacement ellipsoids are drawn at the 50% probability level. Only H atoms connected to O and chiral C atoms are shown for clarity.

The asymmetric unit of the title compound is shown in Fig. 2 ▸. The terminal 3-methyl­oxolan-3-en-2-one unit (C16/O17/C18–C20/O21/C22) is essentially planar with a maximum deviation of 0.0383 (16) Å at atom O17. The oxolane ring (C5/O6/C7–C9) in the fused tricyclic ring system adopts a twist form with puckering parameters of *Q*(2) = 0.350 (3) Å and *φ*(2) = 271.0 (4)°. Atoms C8 and C9 deviate from the plane through the other three atoms by 0.309 (6) and −0.271 (6) Å, respectively. The central seven-membered azepane ring (N1/C2–C5/C9/C10), which is *trans*-fused to the oxolane ring, adopts a twist-chair form with puckering parameters of *Q* = 0.796 (2), *Q*(2) = 0.472 (2) Å, *φ*(2) = 195.0 (3)°, *Q*(3) = 0.641 (2) Å and *φ*(3) = 246.7 (2)°. The pyrrolidine ring (N1/C10–C13) fused to the azepane ring adopts an envelope form with puckering parameters of *Q*(2) = 0.300 (3) Å and *φ*(2) = 251.1 (5)°. The flap atom C11 deviates from the mean plane through the other four atoms by 0.473 (4) Å. The amide moiety (N1/C2/C10/C13/O14) is planar, and atoms N1 and O14 deviate from the mean plane through the other three atoms by 0.028 (2) and −0.035 (4) Å, respectively.

## Supra­molecular features   

**Figure 3:**
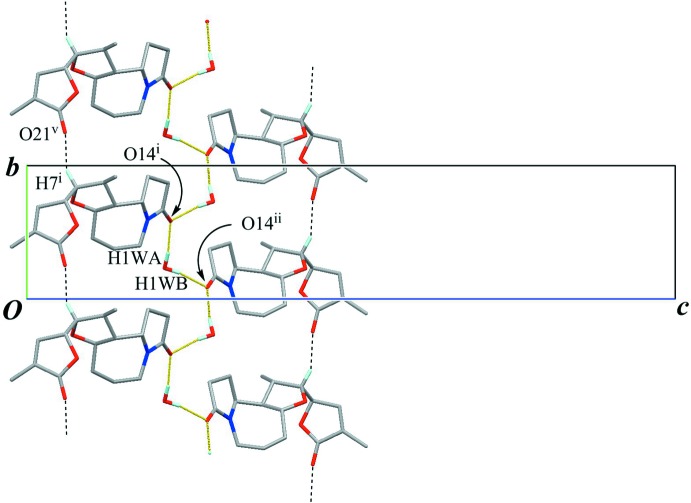
A partial packing diagram viewed down the *a* axis, showing the tape structure running along the *b-*axis direction. Yellow lines indicate the O—H⋯O hydrogen bonds. Black dashed lines indicate C—H⋯O inter­actions. Only H atoms involved in the hydrogen bonds are shown for clarity. [Symmetry codes: (i) *x* − 1, *y*, *z*; (ii) −*x* + 2, *y* − 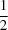, −*z* + 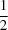; (v) *x* − 1, *y* + 1, *z*.]

**Figure 4:**
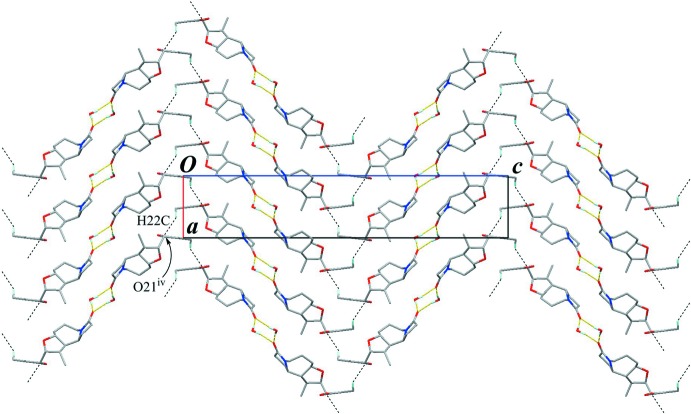
The crystal packing of the title compound, viewed down the *b* axis, showing wavy architectures which consist of the tape structures running along the *b-*axis direction. Yellow lines indicate the O—H⋯O hydrogen bonds. Black dashed lines indicate C—H⋯O inter­actions. Only H atoms involved in the hydrogen bonds are shown for clarity. [Symmetry code: (iv) *x* + 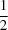, −*y* + 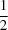, −*z*.]

The crystal packing is stabilized by O—H⋯O hydrogen bonds (O1*W*—H1*WA*⋯O14^i^ and O1*W*—H1*WB*⋯O14^ii^; symmetry codes as in Table 1 ▸) between the water mol­ecule and the amide O atom (Fig. 3 ▸). The amide and water mol­ecules are linked alternately into a tape with a 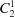
(4) graph-set motif running along the *b-*axis direction. A C—H⋯O inter­action (C7—H7⋯O21^iii^; Table 1 ▸) supports the tape structure, generating a *C*(6) graph-set motif. Furthermore, a weak C—H⋯O inter­action (C22—H22*C*⋯O21^iv^; Table 1 ▸) links the tape structures, extending them into a three-dimensional network (Fig. 4 ▸).

## Database survey   

**Figure 5:**
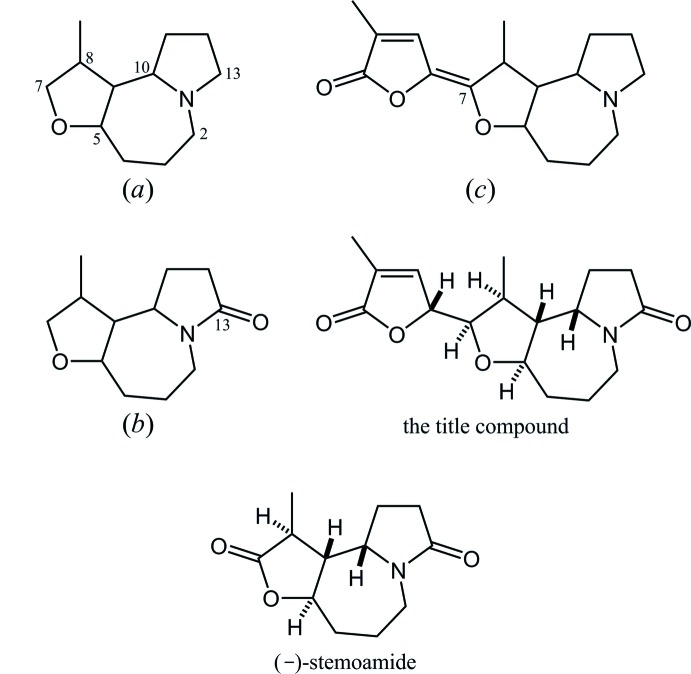
The core structures for database survey; (*a*) 8-methyl-1-aza-6-oxatri­cyclo­[8.3.0.0^5,9^]tridecane, and its (*b*) −13-one and (*c*) 7-(3-methyl-2-oxo-oxolan-3-en-5-yl­idene) derivatives. Structures of the title compound and (–)-stemo­amide are also shown for comparison.

In the Cambridge Structural Database (CSD, Version 5.39, Nov. 2017; Groom *et al.*, 2016 ▸), 19 structures are registered which contain an 8-methyl-1-aza-6-oxatri­cyclo­[8.3.0.0^5,9^]tridecane skeleton, (*a*), *i.e.* the fused tricyclic core related to the title compound (Fig. 5 ▸). These include four structures of its -13-one derivatives, (*b*), with CSD refcodes VATJAC (Kakuta *et al.*, 2003 ▸), KEGYIF (Olivo *et al.*, 2006 ▸), XATFOP (Bates & Sridhar, 2011 ▸) and YAHMIF (Zhang *et al.*, 2011 ▸), and three structures of its 7-(3-methyl-2-oxooxolan-3-en-5-yl­idene) derivatives, (*c*), with refcodes PROTMI (Ishizuka *et al.*, 1972 ▸), PROTOS10 (Irie *et al.*, 1973 ▸) and OJIRII (Kaltenegger *et al.*, 2003 ▸). For the former four structures, the stereochemical and conformational properties, as *trans*-fused furoazepane, relative stereochemistry and conformation of nitro­gen-containing rings, are almost coincident with those of the title compound. On the other hand, the oxolane ring shows an envelope form in these four structures rather than a twist form as in the title compound. KEGYIF (space group *P*2_1_) is the natural alkaloid (–)-stemo­amide, which is a -7,13-dione derivative of (*a*), and XATFOP (space group *P*2_1_/*n*) is its racemate.

## Synthesis and crystallization   

The title compound was afforded in a synthetic study of saxorumamide and isosaxorumamide, from ethyl 4-bromo­butano­ate and a siloxypyrrole analogue (Yoritate *et al.*, 2017 ▸). The stereochemistry was controlled at the first step of the synthesis by enanti­oselective alkynylation according to the reported conditions (Trost *et al.*, 2006 ▸, 2012 ▸), and confirmed with HPLC analysis (>98% ee). The (–)-stemo­amide was provided as a tricyclic core inter­mediate, and its structure and relative and absolute configurations were identical with those reported (Lin *et al.*, 1992 ▸). Purification was carried out by silica gel column chromatography, and pale-yellow crystals were obtained from an EtOAc/hexane mixed solvent (9:1) under a hexane-saturated atmosphere by slow evaporation at ambient temperature, m.p. 466–467 K. [*α*]_D_^23^ – 37.9 (*c* 0.100, CHCl_3_). HRMS (ESI) *m*/*z* calculated for C_17_H_24_NO_4_^+^ [*M* + H]^+^: 306.1705; found: 306.1703.

## Refinement   

Crystal data, data collection and structure refinement details are summarized in Table 2 ▸. C-bound H atoms were positioned geometrically with C—H = 0.95–1.00 Å, and constrained to ride on their parent atoms with *U*_iso_(H) = 1.2*U*_eq_(C) or 1.5*U*_eq_(methyl C). Water H atoms were located in a difference-Fourier map, and then refined freely with *U*_iso_(H) = 1.5*U*_eq_(O), and with distance restraints of O—H = 0.84 (2) Å and H⋯H = 1.33 (4) Å.

## Supplementary Material

Crystal structure: contains datablock(s) global, I. DOI: 10.1107/S2056989018004425/is5494sup1.cif

Structure factors: contains datablock(s) I. DOI: 10.1107/S2056989018004425/is5494Isup2.hkl

CCDC reference: 1830268

Additional supporting information:  crystallographic information; 3D view; checkCIF report

## Figures and Tables

**Table 1:** Hydrogen-bond geometry (Å, °)

*D*—H⋯*A*	*D*—H	H⋯*A*	*D*⋯*A*	*D*—H⋯*A*
O1*W*—H1*WA*⋯O14^i^	0.86 (2)	2.02 (2)	2.874 (3)	173 (3)
O1*W*—H1*WB*⋯O14^ii^	0.86 (2)	1.99 (2)	2.835 (3)	167 (3)
C7—H7⋯O21^iii^	1.00	2.47	3.254 (3)	135
C22—H22*C*⋯O21^iv^	0.98	2.58	3.407 (3)	142

Symmetry codes: (i) 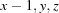; (ii) 
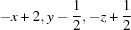
; (iii) 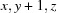; (iv) 
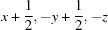
.

**Table 2:** Experimental details

Crystal data	
Chemical formula	C_17_H_23_NO_4_·H_2_O
*M*_r_	323.38
Crystal system, space group	Orthorhombic, *P*2_1_2_1_2_1_
Temperature (K)	90
*a*, *b*, *c* (Å)	6.6180 (3), 7.1197 (3), 34.7351 (15)
*V* (Å^3^)	1636.65 (12)
*Z*	4
Radiation type	Mo *K*α
μ (mm^−1^)	0.10
Crystal size (mm)	0.23 × 0.20 × 0.18

Data collection	
Diffractometer	Bruker D8 Venture
Absorption correction	Multi-scan (*SADABS*; Bruker, 2016 ▸)
*T*_min_, *T*_max_	0.98, 0.98
No. of measured, independent and observed [*I* > 2σ(*I*)] reflections	29644, 2879, 2759
*R*_int_	0.047
(sin θ/λ)_max_ (Å^−1^)	0.595

Refinement	
*R*[*F* ^2^ > 2σ(*F* ^2^)], *wR*(*F* ^2^), *S*	0.033, 0.069, 1.00
No. of reflections	2879
No. of parameters	216
No. of restraints	3
H-atom treatment	H atoms treated by a mixture of independent and constrained refinement
Δρ_max_, Δρ_min_ (e Å^−3^)	0.21, −0.19

Computer programs: *APEX3* (Bruker, 2016 ▸), *SAINT* (Bruker, 2016 ▸), *SHELXT2014/5* (Sheldrick, 2015*a* ▸), *SHELXL2014/7* (Sheldrick, 2015*b* ▸), *Mercury* (Macrae *et al.*, 2008 ▸), *publCIF* (Westrip, 2010 ▸) and *PLATON* (Spek, 2009 ▸).

## supplementary crystallographic information

### Crystal data

**Table:**

C_17_H_23_NO_4_·H_2_O	*D*_x_ = 1.312 Mg m^−^^3^
*M**_r_* = 323.38	Melting point = 466–467 K
Orthorhombic, *P*2_1_2_1_2_1_	Mo *K*α radiation, λ = 0.71073 Å
*a* = 6.6180 (3) Å	Cell parameters from 9946 reflections
*b* = 7.1197 (3) Å	θ = 2.4–25.1°
*c* = 34.7351 (15) Å	µ = 0.10 mm^−^^1^
*V* = 1636.65 (12) Å^3^	*T* = 90 K
*Z* = 4	Prism, pale yellow
*F*(000) = 696	0.23 × 0.20 × 0.18 mm

### Data collection

**Table:**

Bruker D8 Venture diffractometer	2879 independent reflections
Radiation source: fine-focus sealed tube	2759 reflections with *I* > 2σ(*I*)
Multilayered confocal mirror monochromator	*R*_int_ = 0.047
Detector resolution: 7.4074 pixels mm^-1^	θ_max_ = 25.0°, θ_min_ = 2.4°
φ and ω scans	*h* = −7→7
Absorption correction: multi-scan (*SADABS*; Bruker, 2016)	*k* = −8→8
*T*_min_ = 0.98, *T*_max_ = 0.98	*l* = −41→41
29644 measured reflections	

### Refinement

**Table:**

Refinement on *F*^2^	Primary atom site location: structure-invariant direct methods
Least-squares matrix: full	Secondary atom site location: difference Fourier map
*R*[*F*^2^ > 2σ(*F*^2^)] = 0.033	Hydrogen site location: mixed
*wR*(*F*^2^) = 0.069	H atoms treated by a mixture of independent and constrained refinement
*S* = 1.00	*w* = 1/[σ^2^(*F*_o_^2^) + 1.385*P*] where *P* = (*F*_o_^2^ + 2*F*_c_^2^)/3
2879 reflections	(Δ/σ)_max_ < 0.001
216 parameters	Δρ_max_ = 0.21 e Å^−^^3^
3 restraints	Δρ_min_ = −0.19 e Å^−^^3^

### Special details

**Table:**

Experimental. IR (film): 2925, 2855, 1756, 1667, 1455, 1261, 1083, 802 cm^-1^; ^1^H NMR (500 MHz, CDCl_3_): *δ* (p.p.m.) 6.97 (dq, *J* = 1.7, 1.4 Hz, 1H; H20), 4.89–4.86 (m, 1H; H16), 4.22 (dd, *J* = 8.3, 0.9 Hz, 1H; H7), 4.07–4.01 (m, 1H; H2B), 3.92 (ddd, *J* = 10.9, 6.9, 6.3 Hz, 1H; H10), 3.73 (ddd, *J* = 10.2, 9.7, 2.9 Hz, 1H; H5), 2.72–2.65 (m, 1H; H2A), 2.59–2.51 (m, 1H; H8), 2.47 (ddd, *J* = 12.0, 9.7, 6.9 Hz, 1H; H9), 2.42–2.31 (m, 2H; H12AB), 2.00–1.92 (m, 2H; H4A & H11A), 1.95 (dd, *J* = 1.7, 1.4 Hz, 3H; H22ABC), 1.74–1.59 (m, 2H; H3B & H11B), 1.45–1.28 (m, 2H; H3A & H4B), 1.24 (d, *J* = 6.6 Hz, 3H; H15ABC) ^13^C NMR (125 MHz, CDCl_3_): *δ* (p.p.m.) 174.7 (C; C18), 174.1 (C; C13), 146.7 (CH; C19), 131.1 (C; C20), 80.6, 80.3, 80.0 (CH x3; C16, C7 & C5), 56.3 (CH; C10), 51.3 (CH; C9), 40.5 (CH_2_; C2), 38.5 (CH; C8), 35.1 (CH_2_; C12), 31.0 (CH_2_; C11), 25.9 (CH_2_; C4), 22.1 (CH_2_; C3), 13.1 (CH_3_; C22), 10.9 (CH_3_; C15)
Geometry. All esds (except the esd in the dihedral angle between two l.s. planes) are estimated using the full covariance matrix. The cell esds are taken into account individually in the estimation of esds in distances, angles and torsion angles; correlations between esds in cell parameters are only used when they are defined by crystal symmetry. An approximate (isotropic) treatment of cell esds is used for estimating esds involving l.s. planes.
Refinement. Refinement of *F*^2^ against ALL reflections. The weighted *R*-factor *wR* and goodness of fit *S* are based on *F*^2^, conventional *R*-factors *R* are based on *F*, with *F* set to zero for negative *F*^2^. The threshold expression of *F*^2^ > 2*σ*(*F*^2^) is used only for calculating *R*-factors(gt) *etc.* and is not relevant to the choice of reflections for refinement. *R*-factors based on *F*^2^ are statistically about twice as large as those based on *F*, and *R*-factors based on ALL data will be even larger.

### Fractional atomic coordinates and isotropic or equivalent isotropic displacement parameters (Å^2^)

**Table:**

	*x*	*y*	*z*	*U*_iso_*/*U*_eq_	
N1	1.0068 (3)	0.5967 (3)	0.18662 (5)	0.0148 (4)	
C2	1.0057 (4)	0.4003 (3)	0.17441 (6)	0.0163 (5)	
H2A	0.8647	0.3539	0.1739	0.02*	
H2B	1.0817	0.3241	0.1933	0.02*	
C3	1.0999 (4)	0.3752 (4)	0.13469 (7)	0.0174 (5)	
H3A	1.2346	0.4357	0.1346	0.021*	
H3B	1.1204	0.2393	0.13	0.021*	
C4	0.9764 (4)	0.4558 (3)	0.10156 (7)	0.0160 (5)	
H4A	1.0537	0.4403	0.0774	0.019*	
H4B	0.8499	0.3825	0.099	0.019*	
C5	0.9231 (4)	0.6613 (3)	0.10638 (6)	0.0138 (5)	
H5	1.0485	0.7338	0.1126	0.017*	
O6	0.8410 (3)	0.7277 (2)	0.07027 (4)	0.0169 (4)	
C7	0.6625 (4)	0.8368 (3)	0.07655 (7)	0.0165 (5)	
H7	0.678	0.9594	0.0628	0.02*	
C8	0.6556 (4)	0.8755 (3)	0.11996 (7)	0.0156 (5)	
H8	0.7432	0.9872	0.1251	0.019*	
C9	0.7626 (4)	0.7031 (3)	0.13668 (6)	0.0129 (5)	
H9	0.6641	0.5966	0.1368	0.015*	
C10	0.8418 (4)	0.7289 (3)	0.17783 (6)	0.0144 (5)	
H10	0.7283	0.7068	0.1963	0.017*	
C11	0.9401 (4)	0.9177 (3)	0.18706 (7)	0.0171 (5)	
H11A	0.8389	1.0086	0.1967	0.021*	
H11B	1.0069	0.9711	0.164	0.021*	
C12	1.0951 (4)	0.8701 (4)	0.21821 (7)	0.0186 (6)	
H12A	1.2148	0.9531	0.2164	0.022*	
H12B	1.0354	0.8817	0.2442	0.022*	
C13	1.1500 (4)	0.6692 (3)	0.20942 (7)	0.0165 (5)	
O14	1.2991 (3)	0.5844 (3)	0.22177 (5)	0.0233 (4)	
C15	0.4482 (4)	0.9164 (4)	0.13680 (7)	0.0218 (6)	
H15A	0.382	1.0147	0.1216	0.033*	
H15B	0.4629	0.9589	0.1635	0.033*	
H15C	0.366	0.802	0.1362	0.033*	
C16	0.4817 (4)	0.7338 (3)	0.05958 (6)	0.0180 (5)	
H16	0.3564	0.8095	0.0639	0.022*	
O17	0.4601 (3)	0.5530 (2)	0.07769 (5)	0.0184 (4)	
C18	0.4827 (4)	0.4170 (4)	0.05060 (7)	0.0196 (5)	
C19	0.5050 (4)	0.5060 (4)	0.01261 (7)	0.0207 (6)	
C20	0.5018 (4)	0.6892 (4)	0.01778 (7)	0.0203 (6)	
H20	0.5109	0.7799	−0.0022	0.024*	
O21	0.4813 (3)	0.2530 (3)	0.05902 (5)	0.0289 (5)	
C22	0.5281 (4)	0.3907 (4)	−0.02303 (7)	0.0292 (7)	
H22A	0.5321	0.4736	−0.0455	0.044*	
H22B	0.4134	0.3044	−0.0253	0.044*	
H22C	0.654	0.3185	−0.0216	0.044*	
O1W	0.5010 (4)	0.2300 (3)	0.21248 (6)	0.0424 (6)	
H1WA	0.440 (5)	0.334 (4)	0.2174 (10)	0.064*	
H1WB	0.549 (6)	0.198 (5)	0.2345 (7)	0.064*	

### Atomic displacement parameters (Å^2^)

**Table:**

	*U*^11^	*U*^22^	*U*^33^	*U*^12^	*U*^13^	*U*^23^
N1	0.0184 (10)	0.0129 (10)	0.0131 (9)	0.0011 (10)	−0.0012 (9)	0.0001 (8)
C2	0.0202 (12)	0.0116 (11)	0.0170 (11)	−0.0013 (12)	−0.0012 (11)	0.0025 (9)
C3	0.0188 (13)	0.0137 (12)	0.0197 (13)	0.0027 (11)	−0.0005 (11)	−0.0013 (10)
C4	0.0149 (13)	0.0179 (13)	0.0152 (11)	0.0027 (11)	0.0027 (11)	−0.0030 (10)
C5	0.0147 (12)	0.0168 (12)	0.0098 (11)	−0.0005 (10)	−0.0003 (10)	0.0009 (9)
O6	0.0151 (9)	0.0225 (9)	0.0133 (8)	0.0052 (8)	0.0010 (7)	0.0030 (7)
C7	0.0147 (12)	0.0161 (12)	0.0186 (12)	0.0030 (11)	0.0018 (10)	0.0049 (10)
C8	0.0164 (12)	0.0120 (12)	0.0184 (12)	0.0001 (11)	−0.0021 (10)	0.0000 (10)
C9	0.0123 (11)	0.0130 (12)	0.0134 (11)	0.0000 (10)	0.0018 (10)	−0.0001 (10)
C10	0.0157 (12)	0.0146 (12)	0.0130 (11)	0.0009 (11)	0.0038 (10)	0.0002 (10)
C11	0.0204 (13)	0.0133 (12)	0.0177 (12)	0.0015 (11)	0.0027 (10)	−0.0031 (10)
C12	0.0230 (13)	0.0186 (13)	0.0141 (12)	−0.0010 (11)	0.0010 (11)	−0.0045 (10)
C13	0.0220 (14)	0.0183 (13)	0.0092 (11)	−0.0005 (12)	0.0011 (10)	0.0023 (10)
O14	0.0257 (10)	0.0215 (9)	0.0227 (9)	0.0035 (9)	−0.0099 (8)	−0.0002 (8)
C15	0.0203 (13)	0.0235 (13)	0.0217 (13)	0.0057 (12)	−0.0004 (11)	−0.0050 (11)
C16	0.0171 (13)	0.0193 (12)	0.0177 (11)	0.0038 (11)	0.0002 (11)	0.0030 (10)
O17	0.0194 (9)	0.0202 (9)	0.0157 (8)	−0.0035 (8)	−0.0002 (7)	0.0015 (7)
C18	0.0139 (12)	0.0230 (14)	0.0220 (13)	0.0017 (12)	−0.0053 (11)	−0.0009 (11)
C19	0.0133 (12)	0.0297 (14)	0.0190 (12)	0.0060 (13)	−0.0038 (11)	−0.0020 (11)
C20	0.0143 (13)	0.0297 (14)	0.0169 (12)	0.0007 (13)	−0.0022 (11)	0.0058 (10)
O21	0.0334 (11)	0.0210 (10)	0.0324 (10)	0.0006 (10)	−0.0099 (10)	0.0010 (8)
C22	0.0267 (15)	0.0374 (16)	0.0234 (14)	0.0100 (15)	−0.0053 (12)	−0.0085 (12)
O1W	0.0562 (15)	0.0365 (12)	0.0345 (11)	0.0201 (13)	−0.0228 (12)	−0.0136 (10)

### Geometric parameters (Å, º)

**Table:**

N1—C13	1.338 (3)	C10—H10	1.0
N1—C2	1.461 (3)	C11—C12	1.529 (3)
N1—C10	1.474 (3)	C11—H11A	0.99
C2—C3	1.525 (3)	C11—H11B	0.99
C2—H2A	0.99	C12—C13	1.507 (3)
C2—H2B	0.99	C12—H12A	0.99
C3—C4	1.524 (3)	C12—H12B	0.99
C3—H3A	0.99	C13—O14	1.234 (3)
C3—H3B	0.99	C15—H15A	0.98
C4—C5	1.515 (3)	C15—H15B	0.98
C4—H4A	0.99	C15—H15C	0.98
C4—H4B	0.99	C16—O17	1.440 (3)
C5—O6	1.446 (3)	C16—C20	1.492 (3)
C5—C9	1.525 (3)	C16—H16	1.0
C5—H5	1.0	O17—C18	1.359 (3)
O6—C7	1.431 (3)	C18—O21	1.204 (3)
C7—C16	1.522 (4)	C18—C19	1.471 (3)
C7—C8	1.534 (3)	C19—C20	1.317 (4)
C7—H7	1.0	C19—C22	1.493 (3)
C8—C15	1.521 (3)	C20—H20	0.95
C8—C9	1.531 (3)	C22—H22A	0.98
C8—H8	1.0	C22—H22B	0.98
C9—C10	1.533 (3)	C22—H22C	0.98
C9—H9	1.0	O1W—H1WA	0.86 (2)
C10—C11	1.528 (3)	O1W—H1WB	0.86 (2)
			
C13—N1—C2	123.0 (2)	C11—C10—C9	116.5 (2)
C13—N1—C10	113.62 (19)	N1—C10—H10	108.9
C2—N1—C10	123.2 (2)	C11—C10—H10	108.9
N1—C2—C3	111.90 (19)	C9—C10—H10	108.9
N1—C2—H2A	109.2	C10—C11—C12	103.9 (2)
C3—C2—H2A	109.2	C10—C11—H11A	111.0
N1—C2—H2B	109.2	C12—C11—H11A	111.0
C3—C2—H2B	109.2	C10—C11—H11B	111.0
H2A—C2—H2B	107.9	C12—C11—H11B	111.0
C4—C3—C2	114.8 (2)	H11A—C11—H11B	109.0
C4—C3—H3A	108.6	C13—C12—C11	103.2 (2)
C2—C3—H3A	108.6	C13—C12—H12A	111.1
C4—C3—H3B	108.6	C11—C12—H12A	111.1
C2—C3—H3B	108.6	C13—C12—H12B	111.1
H3A—C3—H3B	107.5	C11—C12—H12B	111.1
C5—C4—C3	113.9 (2)	H12A—C12—H12B	109.1
C5—C4—H4A	108.8	O14—C13—N1	125.7 (2)
C3—C4—H4A	108.8	O14—C13—C12	125.9 (2)
C5—C4—H4B	108.8	N1—C13—C12	108.4 (2)
C3—C4—H4B	108.8	C8—C15—H15A	109.5
H4A—C4—H4B	107.7	C8—C15—H15B	109.5
O6—C5—C4	107.90 (18)	H15A—C15—H15B	109.5
O6—C5—C9	105.84 (19)	C8—C15—H15C	109.5
C4—C5—C9	115.3 (2)	H15A—C15—H15C	109.5
O6—C5—H5	109.2	H15B—C15—H15C	109.5
C4—C5—H5	109.2	O17—C16—C20	104.1 (2)
C9—C5—H5	109.2	O17—C16—C7	109.86 (19)
C7—O6—C5	110.83 (17)	C20—C16—C7	114.2 (2)
O6—C7—C16	109.18 (19)	O17—C16—H16	109.5
O6—C7—C8	105.77 (19)	C20—C16—H16	109.5
C16—C7—C8	116.4 (2)	C7—C16—H16	109.5
O6—C7—H7	108.4	C18—O17—C16	108.90 (17)
C16—C7—H7	108.4	O21—C18—O17	121.5 (2)
C8—C7—H7	108.4	O21—C18—C19	129.5 (2)
C15—C8—C9	115.1 (2)	O17—C18—C19	109.0 (2)
C15—C8—C7	116.1 (2)	C20—C19—C18	107.6 (2)
C9—C8—C7	102.43 (19)	C20—C19—C22	131.2 (2)
C15—C8—H8	107.6	C18—C19—C22	121.2 (2)
C9—C8—H8	107.6	C19—C20—C16	110.2 (2)
C7—C8—H8	107.6	C19—C20—H20	124.9
C5—C9—C8	102.52 (19)	C16—C20—H20	124.9
C5—C9—C10	115.4 (2)	C19—C22—H22A	109.5
C8—C9—C10	114.6 (2)	C19—C22—H22B	109.5
C5—C9—H9	108.0	H22A—C22—H22B	109.5
C8—C9—H9	108.0	C19—C22—H22C	109.5
C10—C9—H9	108.0	H22A—C22—H22C	109.5
N1—C10—C11	101.69 (19)	H22B—C22—H22C	109.5
N1—C10—C9	111.73 (18)	H1WA—O1W—H1WB	103 (3)
			
C13—N1—C2—C3	−96.5 (3)	C5—C9—C10—C11	77.7 (3)
C10—N1—C2—C3	89.0 (3)	C8—C9—C10—C11	−41.0 (3)
N1—C2—C3—C4	−69.5 (3)	N1—C10—C11—C12	−28.7 (2)
C2—C3—C4—C5	54.7 (3)	C9—C10—C11—C12	−150.4 (2)
C3—C4—C5—O6	169.37 (19)	C10—C11—C12—C13	28.6 (2)
C3—C4—C5—C9	−72.6 (3)	C2—N1—C13—O14	2.5 (4)
C4—C5—O6—C7	134.6 (2)	C10—N1—C13—O14	177.4 (2)
C9—C5—O6—C7	10.7 (3)	C2—N1—C13—C12	−176.1 (2)
C5—O6—C7—C16	−113.8 (2)	C10—N1—C13—C12	−1.1 (3)
C5—O6—C7—C8	12.1 (3)	C11—C12—C13—O14	163.8 (2)
O6—C7—C8—C15	−155.9 (2)	C11—C12—C13—N1	−17.7 (2)
C16—C7—C8—C15	−34.4 (3)	O6—C7—C16—O17	59.7 (2)
O6—C7—C8—C9	−29.6 (2)	C8—C7—C16—O17	−59.9 (3)
C16—C7—C8—C9	91.8 (2)	O6—C7—C16—C20	−56.8 (3)
O6—C5—C9—C8	−28.7 (2)	C8—C7—C16—C20	−176.4 (2)
C4—C5—C9—C8	−147.9 (2)	C20—C16—O17—C18	4.8 (3)
O6—C5—C9—C10	−153.92 (19)	C7—C16—O17—C18	−117.8 (2)
C4—C5—C9—C10	86.9 (3)	C16—O17—C18—O21	176.1 (3)
C15—C8—C9—C5	161.9 (2)	C16—O17—C18—C19	−4.3 (3)
C7—C8—C9—C5	35.0 (2)	O21—C18—C19—C20	−178.6 (3)
C15—C8—C9—C10	−72.4 (3)	O17—C18—C19—C20	1.9 (3)
C7—C8—C9—C10	160.7 (2)	O21—C18—C19—C22	0.8 (4)
C13—N1—C10—C11	19.3 (2)	O17—C18—C19—C22	−178.7 (2)
C2—N1—C10—C11	−165.7 (2)	C18—C19—C20—C16	1.2 (3)
C13—N1—C10—C9	144.3 (2)	C22—C19—C20—C16	−178.1 (3)
C2—N1—C10—C9	−40.7 (3)	O17—C16—C20—C19	−3.7 (3)
C5—C9—C10—N1	−38.5 (3)	C7—C16—C20—C19	116.1 (3)
C8—C9—C10—N1	−157.2 (2)		

### Hydrogen-bond geometry (Å, º)

**Table:**

*D*—H···*A*	*D*—H	H···*A*	*D*···*A*	*D*—H···*A*
O1*W*—H1*WA*···O14^i^	0.86 (2)	2.02 (2)	2.874 (3)	173 (3)
O1*W*—H1*WB*···O14^ii^	0.86 (2)	1.99 (2)	2.835 (3)	167 (3)
C7—H7···O21^iii^	1.00	2.47	3.254 (3)	135
C22—H22*C*···O21^iv^	0.98	2.58	3.407 (3)	142

Symmetry codes: (i) *x*−1, *y*, *z*; (ii) −*x*+2, *y*−1/2, −*z*+1/2; (iii) *x*, *y*+1, *z*; (iv) *x*+1/2, −*y*+1/2, −*z*.
